# The effect of deep or sustained remission on maintenance of remission after dose reduction or withdrawal of etanercept in patients with rheumatoid arthritis

**DOI:** 10.1186/s13075-019-1937-4

**Published:** 2019-07-05

**Authors:** Yoshiya Tanaka, Josef S. Smolen, Heather Jones, Annette Szumski, Lisa Marshall, Paul Emery

**Affiliations:** 1 The First Department of Internal Medicine, University of Occupational and Environmental Health, Japan, 1-1 Iseigaoka, Yahata-nishi, Kitakyushu, 807-8555 Japan; 2 Medical University of Vienna, Vienna, Austria; 3 Pfizer, Collegeville, PA USA; 4 Syneos Health, Princeton, NJ USA; 5 Leeds Biomedical Research Centre, Leeds Institute of Rheumatic and Musculoskeletal Medicine, University of Leeds, Leeds, UK

**Keywords:** Rheumatoid arthritis, Anti-TNF, Etanercept, Remission, Dose reduction, Withdrawal, DAS28, ACR/EULAR Boolean, CDAI

## Abstract

**Background:**

Biologic disease-modifying antirheumatic drugs (bDMARDs) are important options for managing rheumatoid arthritis (RA). Once patients achieve disease control, clinicians may consider dose reduction or withdrawal of the bDMARD. Results from published studies indicate that some patients will maintain remission; however, others will flare. We analyzed data from three etanercept down-titration studies in patients with RA to determine what extent of remission provides the greatest predictability of maintaining remission following dose reduction or discontinuation.

**Methods:**

Patients with moderate to severe RA from the PRESERVE, PRIZE, and Treat-to-Target (T2T) randomized controlled trials were included. We determined the proportion of patients achieving remission with etanercept at the last time point in the induction period, and sustained remission (last two time points), according to the Disease Activity Score 28-joints (DAS28), the American College of Rheumatology (ACR)/European League Against Rheumatism (EULAR) Boolean criteria, and the clinical disease activity index (CDAI). We also calculated the proportion achieving DAS28 deep remission (DAS28 ≤ 1.98), sustained deep remission (last two time points), and low disease activity (LDA), and LDA according to the CDAI. Then, we evaluated whether they maintained remission or LDA following etanercept dose reduction or withdrawal.

**Results:**

Patients achieving sustained and/or deep remission were more likely than patients achieving remission or LDA to maintain remission/LDA after etanercept dose reduction or withdrawal. In PRESERVE, the proportions of patients with DAS28 sustained deep remission, deep remission, sustained remission, remission, and LDA who maintained remission following etanercept dose reduction were 81%, 67%, 58%, 56%, and 36%, respectively, *P* < 0.001 for trend. In PRESERVE, this trend was significant when etanercept was discontinued and when ACR/EULAR Boolean and CDAI remission criteria were used. Although some sample sizes were small, the PRIZE and T2T studies also demonstrated response trends according to ACR/EULAR Boolean and CDAI remission criteria, and T2T demonstrated response trends according to DAS28.

**Conclusions:**

These results suggest that patients achieving disease control according to a stringent definition, such as sustained ACR/EULAR Boolean or CDAI remission, or a new definition of sustained deep remission by DAS28, have a higher probability of remaining in remission or LDA following etanercept dose reduction or withdrawal.

**Trial registration:**

PRESERVE: ClinicalTrials.gov identifier: NCT00565409, registered 30 November 2007; PRIZE: ClinicalTrials.gov identifier: NCT00913458, registered 4 June 2009; T2T: ClinicalTrials.gov identifier: NCT01578850, registered 17 April 2012

**Electronic supplementary material:**

The online version of this article (10.1186/s13075-019-1937-4) contains supplementary material, which is available to authorized users.

## Background

Treatment guidelines for rheumatoid arthritis (RA) recommend intensive therapy targeting clinical remission early in the disease course, when patients have a higher likelihood of responding to treatment [1–5]. This treatment target has been shown to correlate with better patient-reported outcomes, greater productivity, and lower overall healthcare costs than achieving low disease activity (LDA) [6, 7].

Biologic disease-modifying antirheumatic drugs (bDMARDs) are important therapeutic options in this treat-to-target strategy. However, once patients achieve disease control, clinicians may consider dose reduction or withdrawal of the bDMARD out of concerns for infection risk, dose-dependent adverse events, or treatment cost [8, 9]. As noted in review articles, studies evaluating down-titration of bDMARDs have used various protocols with respect to baseline disease activity and the length of time that RA symptoms were under control before dose reduction or withdrawal of the bDMARD [8–16]. For example, studies have required patients to have LDA according to Disease Activity Score 28-joint count (DAS28 ≤ 3.2) for 6 months [17, 18] or DAS28 remission (DAS28 < 2.6) for 6 months [19, 20] or 12 months [21], LDA according to DAS28 or clinician judgment for 3 months [22, 23], moderate to good European League Against Rheumatism (EULAR) response at month 6 [24], or remission according to the clinical disease activity index (CDAI) at month 6 [25].

Published success rates following bDMARD dose reduction or withdrawal vary widely; in many studies, approximately 40–60% of patients were able to maintain remission or LDA, while the remaining patients experienced a flare [8–16]. A higher percentage of patients tend to flare if the bDMARD is withdrawn rather than decreased in dose [1, 8–11, 13].

According to treatment guidelines issued by EULAR, the Asia Pacific League of Associations for Rheumatology (APLAR), and the American College of Rheumatology (ACR), clinicians may consider tapering the bDMARD when a patient is in persistent remission [1–3]. Unfortunately, it is not possible to predict with 100% certainty which patients will maintain remission following dose reduction or withdrawal. This is an ongoing area of research; one study with a published protocol, PREDICTRA, is evaluating which disease and patient characteristics may be useful in predicting the outcome of tapering a bDMARD [26]. Evidence from published studies to date suggests that predictors for successful tapering include early RA, remission duration of at least 6 months, and certain clinical signs such as normal levels of inflammatory markers and/or absence of synovitis on ultrasound [8, 27]. One study that evaluated the relationship between psychological factors and flare with dose decrease found that baseline scores on the 36-Item Short Form Survey Mental Health component and the DAS28 were significant predictors of flare [28].

Analyses from two open-label, nonrandomized clinical studies (RRR and HONOR) evaluated whether achieving DAS28 remission according to a lower cut-off value than 2.6 (referred to as deep remission) would enable more patients to maintain DAS28 remission or LDA following bDMARD discontinuation [29, 30]. In the RRR trial (*n* = 102 patients), the significant DAS28 cut-off point was determined to be < 2.225; in the HONOR trial (*n* = 52 patients), it was 1.98 [29, 30].

However, measurement of remission with the DAS28 has been criticized since patients can achieve DAS28 remission but still have active disease, such as tender and swollen joints [31]. Additionally, studies have shown that if the DAS28 cut-points are used with therapies that directly affect the acute-phase response, such as interleukin (IL)-6 inhibitors or Janus kinase (JAK) inhibitors, significant residual disease activity is likely to remain [32–34]. This is the case even if the more stringent remission cut-points of DAS28 (calculated using C-reactive protein [CRP]) < 1.9 and DAS28 (calculated using erythrocyte sedimentation rate [ESR]) < 2.2 are used [32, 35]. Therefore, alternative outcome measures, such as the ACR/EULAR index-based or Boolean remission criteria, the CDAI, or the Simplified Disease Activity Index (SDAI), should also be considered when evaluating predictors for maintaining remission following bDMARD dose reduction or discontinuation.

Based on results from the literature, we hypothesized that achieving sustained remission (ACR/EULAR Boolean or CDAI remission criteria) or sustained deep remission (DAS28 remission criteria) will increase the likelihood of maintaining remission or LDA following dose reduction or discontinuation of bDMARD therapy. We conducted a post hoc analysis of data from three etanercept down-titration studies in patients with RA to determine what extent of remission will provide the greatest predictability of maintaining remission following dose reduction or discontinuation. The objectives of this analysis were to (1) compare baseline characteristics of patients who achieved sustained deep remission, deep remission, sustained remission, remission, and LDA with full-dose etanercept therapy; (2) determine if the extent of the initial response was predictive of the likelihood of maintaining remission or LDA after the etanercept dose was decreased or discontinued; and (3) compare rates of remission and LDA measured according to the DAS28 criteria, rates of remission measured according to the ACR/EULAR Boolean criteria, and rates of remission and LDA measured according to the CDAI criteria.

## Methods

This analysis included patients with RA from three randomized, controlled clinical trials (PRESERVE, PRIZE, and Treat-to-Target [T2T]) that consisted of an open-label induction period with etanercept 50 mg weekly (period 1) followed by a double-blind maintenance, reduction, or withdrawal period (period 2) [17, 36, 37]. The populations differed among the studies, the patients in PRESERVE had moderate RA disease activity and a mean disease duration of 6.9 years, and the patients in PRIZE and T2T had moderate to high (“moderate-to-severe”) RA disease activity and mean disease durations of 6.5 months and 8.0 years, respectively [17, 36]. Additional details of the study designs are presented in Table 1. Patients were included in this analysis if they completed period 1 and were randomized into period 2.

**Table 1:** Study designs for PRESERVE, PRIZE, and T2T

Study	RA Status	DMARD history	Period 1 design^a^	Criteria to enter period 2	Period 2 design^a^	Primary endpoint
PRESERVE [17]	Moderate (DAS28 > 3.2 and ≤ 5.1)	MTX 15–25 mg QW for 8 weeks prior to screening; bDMARD naive	36 weeks: ETN50 + MTX QW	Sustained LDA (mean DAS28 ≤ 3.2) from weeks 12–36 and DAS28 ≤ 3.2 at week 36	52 weeks: 1. ETN50 + MTX	% patients in the ETN50 + MTX and PBO + MTX groups achieving DAS28 LDA at week 88.^b^ Conditional primary endpoint was DAS28 LDA at week 88 with ETN25 + MTX
					2. ETN25 + MTX	
					3. PBO + MTX, all QW	
PRIZE [37]	Moderate-to-severe (DAS28 > 3.2) early RA (symptom onset ≤ 12 months prior to enrollment)	MTX and bDMARD naive	52 weeks: ETN50 + MTX QW	DAS28 ≤ 3.2 at week 39 and DAS28 < 2.6 at week 52	39 weeks: 1. ETN25 + MTX	% patients with sustained remission (DAS28 < 2.6) at weeks 76 and 91 (no corticosteroids from weeks 52 to 64)^c^
					2. PBO + MTX	
					3. PBO + PBO	
T2T [36]	Moderate-to-severe	Inadequate response to MTX	24 weeks: ETN50 + MTX QW ± other csDMARDs^d^	LDA (DAS28 < 3.2) at week 24	28 weeks: 1. ETN50 + MTX ± other csDMARDs^d^	% patients with DAS28 LDA at week 52 without rescue medication
					2. PBO + MTX ± other csDMARDs^d^	

*bDMARD* biologic disease-modifying antirheumatic drug, *csDMARD* conventional synthetic DMARD, *DAS28* Disease Activity Score 28-joint count, *ETN* etanercept, *LDA* low disease activity, *MTX* methotrexate, *PBO* placebo, *QW* weekly, *RA* rheumatoid arthritis, *T2T* Treat-to-Target

^a^For all studies, period 1 was open-label induction and period 2 was double-blind maintenance or withdrawal

^b^A modified nonresponder imputation analysis was conducted: patients who discontinued due to lack of efficacy were treated as nonresponders

^c^All patients who discontinued were considered nonresponders

^d^Sulfasalazine, hydroxychloroquine, and leflunomide

The three studies were conducted according to the International Conference on Harmonization guidelines for Good Clinical Practice as well as the ethical principles of the Declaration of Helsinki. All patients provided informed consent prior to enrolment, and the institutional review board or independent ethics committee at each participating center reviewed and approved the study protocol and consent forms (see “Acknowledgments” for details).

For all treatment groups in the PRESERVE, PRIZE, and T2T studies, clinical response at the last time point in period 1 was determined according to three different remission criteria: DAS28, ACR/EULAR Boolean, and CDAI. Sustained responses were measured at the last two time points in period 1, which were as follows: PRESERVE—weeks 28 and 36; PRIZE—weeks 39 and 52; and T2T—weeks 16 and 24. In each study, DAS28 was calculated using ESR [38].

Specifically, the following responses, in order of disease activity, were measured at the end of period 1:

- DAS28 remission criteria [38]:
  - Sustained deep remission (deep remission at the last two time points, with deep remission defined as DAS28 ≤ 1.98) [30]
  - Deep remission
  - Sustained remission (remission at the last two time points, with remission defined as DAS28 > 1.98 to < 2.6)
  - Remission
  - LDA (DAS28 ≥ 2.6 to < 3.2; PRESERVE and T2T)
- ACR/EULAR Boolean remission criteria [39]:
  - Sustained Boolean remission (remission at the last two time points, with remission defined as tender joint count (TJC) ≤ 1, swollen joint count (SJC) ≤ 1, C-reactive protein (CRP) ≤ 1 mg/dL, and patient global assessment ≤ 1 on a 0–10 scale)
  - Remission
  - Nonremission
- CDAI remission criteria [40]:
  - Sustained CDAI remission (remission at the last two time points, with remission defined as CDAI 0.0–2.8)
  - Remission
  - LDA (CDAI > 2.8–10.0)
  - Moderate disease activity (CDAI > 10.0–22.0; PRESERVE and T2T)

For the PRIZE study, we did not include DAS28 LDA at the end of period 1 because only patients who achieved remission at the end of period 1 were allowed to enter period 2. Additionally, we did not include CDAI moderate disease activity for PRIZE because there were no patients in this category at the end of period 1.

Then, the proportion of patients maintaining remission or LDA (DAS28 and CDAI criteria only) in period 2 was determined according to each response level in period 1. Period 2 response (remission or LDA) was measured only at the last time point in period 2 (PRESERVE: week 88, PRIZE: week 91, T2T: week 52). Patients with remission or LDA at the last time point in period 2 were considered to have maintained that response following dose decrease or withdrawal of etanercept. However, it is unknown whether those patients were in remission or LDA at other time points in period 2.

Demographic and baseline disease characteristics of the patients in each study were categorized according to the following DAS28 responses at the end of period 1: sustained deep remission, deep remission, sustained remission, remission, and LDA (LDA for PRESERVE and T2T), and also according to the following CDAI responses at the end of period 1: sustained remission, remission, LDA, and moderate disease activity (MDA) (MDA for PRESERVE and T2T).

### Statistical analyses

Trend in baseline characteristics among the period 1 response categories (DAS28 or CDAI) was analyzed using one-way analysis of variance for continuous variables with response categories treated as ordered, and the Cochran-Mantel-Haenszel (CMH) test of linear association for categorical variables. The CMH test was also used to evaluate the trend in proportion of responders (i.e., remission or LDA) in period 2 among period 1 responders, using a last observation carried forward approach within period 2.

We conducted two additional analyses. We determined the proportion of patients with a normal value on the Health Assessment Questionnaire (HAQ ≤ 0.5) at the end of period 1 according to whether or not they achieved sustained DAS28 deep remission at the end of period 1. Finally, we conducted four stepwise predictor analyses and a sensitivity analysis to determine the best subset of significant predictors in maintaining DAS28 remission in period 2. The predictors from period 1 baseline that were included in the analyses were selected because each of them had a significant relationship with period 1 DAS28 response categories. In the first stepwise analysis, the predictors included sustained deep remission, age in 10-year units, gender, HAQ ≤ 0.5, ESR ≤ upper limit of normal (ULN), body mass index (BMI ≤ 18.5, > 18.5 to ≤ 30, > 30), SJC = 0 out of 28, and TJC = 0 out of 28. Additional stepwise predictor analyses included the following predictors: study and period 2 treatment group and/or continuous rather than dichotomous BMI and SJC/TJC.

## Results

### Clinical response in period 1 and baseline characteristics according to clinical response

This analysis includes 600, 598, and 594 patients in the ACR/EULAR Boolean, CDAI, and DAS28 remission criteria calculations, respectively, from the PRESERVE study; 193, 192, and 192 patients, respectively, from the PRIZE study; and 331 patients in all three remission criteria calculations from the T2T study. This variability is due to (1) five patients in PRESERVE with DAS28 values > 3.2 at week 36 and one patient with a missing DAS28 value being excluded from the DAS28 analysis, (2) one patient in PRIZE with DAS28 > 2.6 at week 52 being excluded from the DAS28 analysis, and (3) three patients (two from PRESERVE and one from PRIZE) missing component(s) required for calculating CDAI remission.

Table 2 presents the proportion of patients in each DAS28 response category at the end of period 1, as well as demographics and baseline disease characteristics according to each DAS28 response at the end of period 1. All three studies demonstrated a significant difference in mean age according to response category, with younger age being associated with a better DAS28 response in period 1. Lower values for ESR, DAS28-ESR, and HAQ were associated with a better response in PRESERVE and T2T; as were lower values for TJC and CDAI, and a lower proportion of females in T2T; lower BMI in women in PRESERVE and PRIZE; and lower SJC in PRIZE.

**Table 2:** Demographic and baseline disease characteristics according to DAS28 response category at the end of period 1

	PRESERVE, *N* = 594					PRIZE, *N* = 192				T2T, *N* = 331				
	Sustained deep remission *n* = 133 (22%)	Deep remission *n* = 136 (23%)	Sustained remission *n* = 96 (16%)	Remission *n* = 116 (20%)	LDA *n* = 113 (19%)	Sustained deep remission *n* = 67 (35%)	Deep remission *n* = 40 (21%)	Sustained remission *n* = 39 (20%)	Remission *n* = 46 (24%)	Sustained deep remission *n* = 13 (4%)	Deep remission *n* = 30 (9%)	Sustained remission n = 10 (3%)	Remission *n* = 70 (21%)	LDA *n* = 208 (63%)
Demographic characteristics														
Age, years, mean (SD)	43.6 (12.6)	47.9 (12.1)	47.1 (12.0)	47.9 (12.3)	51.8 (10.3)***	43.7 (14.4)	54.1 (13.7)	49.7 (13.3)	53.3 (13.7)**	41.1 (13.5)	44.4 (14.4)	52.3 (11.5)	44.3 (11.7)	47.8 (12.1)*
Female, *n* (%)	97 (73)	111 (82)	82 (85)	98 (84)	91 (81)	41 (61)	27 (68)	25 (64)	31 (67)	9 (69)	22 (73)	9 (90)	57 (81)	182 (88)*
BMI, kg/m^2^, mean (SD)	25.0 (3.9)	25.5 (4.2)	25.8 (5.3)	25.2 (4.3)	26.4 (5.1)	24.4 (3.5)	26.5 (4.9)	26.3 (5.0)	26.8 (5.0)**	24.4 (4.6)	26.5 (4.7)	23.6 (3.1)	28.4 (5.9)	26.8 (5.3)
BMI, female	24.6 (3.8)	25.3 (4.3)	25.8 (5.4)	24.9 (4.3)	26.7 (5.2)*	23.2 (3.4)	25.5 (4.6)	25.7 (4.4)	25.6 (4.9)*	25.0 (4.6)	26.7 (5.0)	23.5 (3.2)	28.4 (6.0)	26.7 (5.4)
BMI, male	26.3 (4.2)	26.1 (3.8)	25.9 (4.9)	26.7 (4.3)	25.4 (4.6)	26.2 (3.1)	28.7 (4.8)	27.5 (5.9)	29.1 (4.3)	23.2 (5.1)	26.0 (3.8)	24.6	28.5 (5.8)	27.3 (4.9)
Prior treatment^a^														
Corticosteroid(s), *n* (%)	76 (57)	76 (56)	56 (58)	77 (66)	70 (62)	22 (33)	18 (45)	17 (44)	22 (48)	9 (69)	17 (57)	6 (60)	52 (74)	140 (67)
NSAID(s), *n* (%)	98 (74)	103 (76)	68 (71)	91 (78)	85 (75)	47 (70)	27 (68)	27 (69)	29 (63)	11 (85)	19 (63)	6 (60)	40 (57)	144 (69)
DMARD(s),^b^ *n* (%)	37 (28)	41 (30)	18 (19)	29 (25)	23 (20)	11 (16)	11 (28)	5 (13)	6 (13)	4 (31)	10 (33)	2 (20)	38 (54)	65 (31)
Number of DMARD(s)^b,c^ mean (min, max)	1.3 (1, 3)	1.3 (1, 3)	1.2 (1, 3)	1.3 (1, 5)	1.2 (1, 2)	1.0 (1, 1)	1.0 (1, 1)	1.0 (1, 1)	1.0 (1, 1)	1.3 (1, 2)	1.3 (1, 2)	1.0 (1, 1)	1.5 (1, 3)	1.3 (1, 3)
Disease characteristics														
Duration of disease, mean (SD)	6.3 (6.8) years	6.6 (6.2) years	7.3 (6.5) years	7.3 (7.7) years	6.7 (7.1) years	6.7 (2.8) months	7.4 (3.2) months	6.3 (2.6) months	7.0 (3.0) months	7.9 (6.2) years	6.3 (5.6) years	8.0 (4.8) years	8.3 (6.8) years	8.4 (7.5) years
RF+, *n* (%)	96 (72)	94 (69)	77 (80)	84 (72)	78 (69)	44 (66)	25 (63)	18 (46)	24 (52)	9 (69)	23 (77)	9 (90)	59 (84)	175 (84)
aCCP antibody+, *n* (%)	100 (75)	99 (73)	82 (85)	99 (85)	85 (75)	47 (70)	27 (68)	23 (59)	29 (63)	9 (69)	24 (80)	10 (100)	49 (70)	171 (82)
ESR, mm/h, mean (SD)	17.9 (10.6)	20.8 (13.0)	24.4 (12.9)	22.7 (11.8)	22.1 (13.9)**	30.1 (22.3)	36.3 (24.3)	38.4 (20.6)	31.7 (19.1)	25.5 (13.8)	37.6 (21.7)	32.1 (15.1)	43.7 (27.7)	50.8 (24.7)***
CRP, mg/L, mean (SD)	10.7 (13.8)	10.5 (12.1)	11.4 (14.2)	11.8 (14.7)	14.2 (20.4)	13.4 (18.6)	19.8 (27.1)	16.3 (24.3)	13.8 (16.6)	21.5 (18.2)	22.0 (16.7)	14.9 (11.9)	21.1 (19.9)	24.0 (29.3)
Disease activity and patient-reported outcomes, mean (SD)														
TJC (0–28)	4.9 (2.8)	5.2 (2.4)	4.7 (2.8)	4.8 (2.8)	5.5 (3.5)	12.5 (5.8)	13.3 (6.8)	13.4 (6.8)	14.3 (7.2)	10.1 (6.6)	12.3 (6.7)	13.7 (6.4)	14.1 (6.7)	14.3 (6.1)*
SJC (0–28)	3.9 (2.7)	3.8 (2.5)	4.0 (2.7)	3.6 (2.4)	4.4 (3.1)	8.8 (5.0)	11.0 (6.0)	11.1 (6.3)	11.0 (5.2)*	9.5 (5.4)	9.9 (5.9)	10.5 (5.5)	10.7 (5.4)	10.6 (5.5)
DAS28-ESR, mean (SD)	4.2 (0.5)	4.3 (0.4)	4.4 (0.4)	4.4 (0.4)	4.4 (0.5)***	5.6 (1.0)	6.0 (1.1)	6.0 (1.1)	5.9 (1.1)	5.5 (1.3)	6.0 (1.2)	6.1 (0.9)	6.3 (1.1)	6.5 (0.9)***
CDAI (0–76)	17.8 (4.9)	17.4 (4.6)	17.3 (5.2)	17.5 (4.6)	18.9 (5.6)	–	–	–	–	31.2 (15.0)	35.3 (12.5)	38.1 (11.0)	38.6 (12.7)	38.3 (11.4)*
PGA (0–10)	4.1 (1.4)	3.9 (1.3)	4.0 (1.3)	4.2 (1.3)	4.1 (1.2)	5.2 (1.6)	5.8 (1.5)	5.8 (1.8)	5.5 (1.5)	6.1 (1.7)	6.8 (1.3)	7.2 (1.0)	6.9 (1.6)	6.7 (1.3)
HAQ (0–3)	0.9 (0.5)	1.2 (0.6)	1.1 (0.5)	1.1 (0.6)	1.2 (0.5)*	1.1 (0.5)	1.3 (0.6)	1.2 (0.7)	1.1 (0.7)	1.0 (0.8)	1.3 (0.6)	1.3 (0.7)	1.6 (0.7)	1.5 (0.6)**

*aCCP* anti-cyclic citrullinated peptide, *BMI* body mass index, *CDAI* Clinical Disease Activity Index, *CRP* C-reactive protein, *DAS28* Disease Activity Score in 28 joints, *DAS28-ESR* Disease Activity Score in 28 joints calculated with erythrocyte sedimentation rate, *DMARD* disease-modifying antirheumatic drug, *ESR* erythrocyte sedimentation rate, *HAQ* health assessment questionnaire, *LDA* low disease activity, *MTX* methotrexate, *NSAID* nonsteroidal anti-inflammatory drug, *PGA* physician global assessment, *RA* rheumatoid arthritis, *RF* rheumatoid factor, *SD* standard deviation, *SJC* swollen joint count, *TJC* tender joint count

^a^In PRESERVE, “prior” was within 6 months of screening (for DMARDs not including MTX), within 28 days of screening or baseline (for glucocorticoids), or concurrent treatment with ≥ 1 NSAID at baseline; in PRIZE, “prior” was 28 days before screening (for NSAIDs and corticosteroids) and any time before screening (for DMARDs); in T2T, “prior” was 6 months before screening (for MTX and DMARDs,) or 4 weeks before screening (for NSAIDs and corticosteroids)

^b^Conventional DMARDs other than MTX

^c^Mean, minimum, and maximum were calculated only from patients who did have prior DMARD(s)

*P < 0.05 across response categories; **P < 0.01 across response categories; ***P < 0.001 across response categories

The proportion of patients in each CDAI response category at the end of period 1 and demographics and baseline disease characteristics according to each CDAI response are presented in Additional file 1: Table S1. In all three studies, younger age was associated with a better clinical response. In the PRESERVE and PRIZE studies, prior treatment with a corticosteroid was associated with a poorer clinical response, and higher baseline ESR, lower TJC, lower SJC, and lower BMI in women were associated with a better clinical response. In PRESERVE, lower CDAI, physician’s global assessment, and HAQ were each associated with a better clinical response, as were positive laboratory results for rheumatoid factor and anti-cyclic citrullinated peptide antibodies in PRIZE.

The proportion of patients achieving each ACR/EULAR Boolean criteria response at the end of period 1 is provided in Additional file 1: Table S2.

### Predictor analyses

All four stepwise predictor analyses and the sensitivity analysis determined that at the end of period 1, the significant predictors of period 2 DAS28 remission were sustained deep remission, ESR ≤ ULN, SJC = 0, and TJC = 0. Since ESR is included in the DAS28 calculation, this association is expected. Study and treatment group were also determined to be significant predictors. Patients who continued etanercept 50 mg were most likely to have DAS28 remission at the end of period 2, followed by patients who decreased the dose to 25 mg, and lastly by patients who discontinued etanercept. When SJC and TJC were treated as continuous, they were found to be significant as well. HAQ ≤ 0.5, age, gender, and BMI were not significant predictors. The significant results from one of the predictor analyses are presented in Table 3.

**Table 3:** Subset of significant predictors of period 2 DAS28 remission

End of period 1 predictor	Odds ratio (95% CI)	*P* value
SJC28 = 0 vs > 0	2.1 (1.4, 3.0)	< 0.001
TJC28 = 0 vs > 0	1.7 (1.2, 2.5)	0.003
Study		0.008
PRESERVE vs T2T	0.7 (0.4, 1.2)	
PRIZE vs T2T	1.7 (0.8, 3.5)	
Sustained deep remission vs not sustained remission	2.4 (1.5, 3.9)	< 0.001
ESR ≤ ULN vs > ULN	2.7 (1.5, 4.8)	0.001
Treatment		< 0.001
ETN 25 mg + MTX vs PBO + MTX	2.7 (1.7, 4.3)	
ETN 50 mg + MTX vs PBO + MTX	3.3 (2.1, 5.0)	
MTX vs PBO	0.7 (0.3, 1.7)	

The subset of significant predictors was determined using a stepwise model with the following “end of period 1” predictors: sustained deep remission vs not; age in 10-year units; gender; body mass index ≤ 18.5, > 18.5 to ≤ 30, 30; HAQ ≤ 0.5 vs > 0.5; ESR ≤ ULN vs > ULN; SJC28 = 0 vs > 0; TJC28 = 0 vs > 0; study; treatment group

*CI* confidence interval, *ESR* erythrocyte sedimentation rate, *ETN* etanercept, *HAQ* health assessment questionnaire, *MTX* methotrexate, *PBO* placebo, *SJC28* swollen joint count measured using 28 joints, *TJC28* tender joint count measured using 28 joints, *ULN* upper limit of normal

### Clinical response in period 2

#### PRESERVE study

**Fig. 1:**
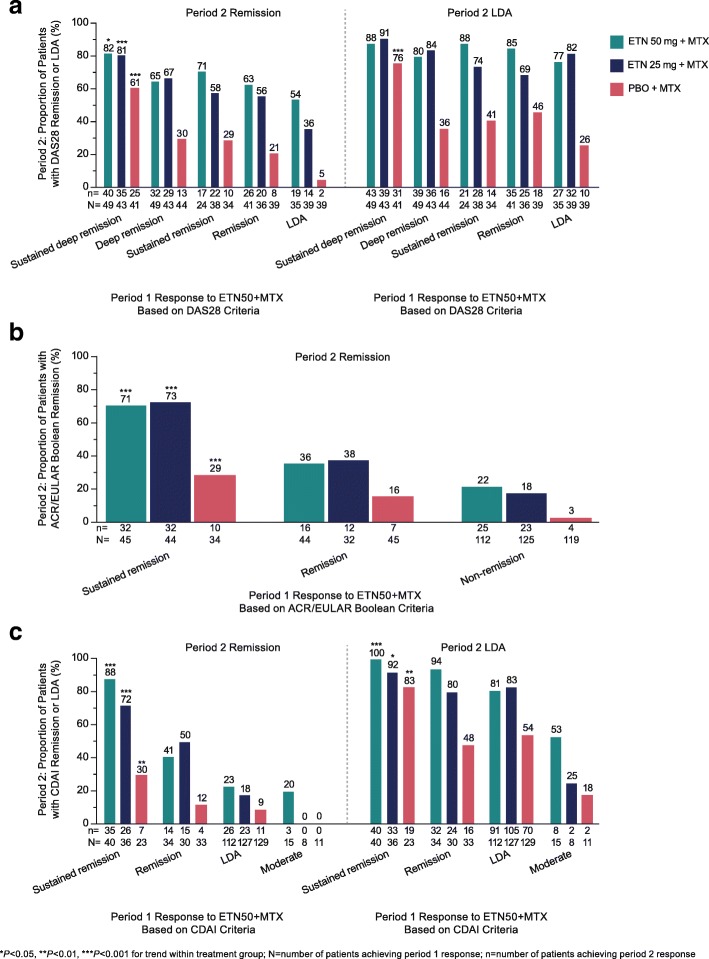
Proportion of patients in the PRESERVE study maintaining remission or LDA in period 2, according to response category in period 1 for the remission criteria of DAS28 (**a**), ACR/EULAR Boolean (**b**), and CDAI (**c**). *ACR* American College of Rheumatology, *CDAI* Clinical Disease Activity Index, *DAS28* Disease Activity Score in 28 joints, *ETN50* etanercept 50 mg, *EULAR* European League Against Rheumatism, *LDA* low disease activity, *MTX* methotrexate, *PBO* placebo

Patients in the PRESERVE study demonstrated significant remission response trends in period 2 for all three measurement criteria in all treatment groups. Patients who achieved DAS28 sustained deep remission in period 1 and then discontinued etanercept were more likely to maintain DAS28 remission in period 2 (25/41, 61%) than those who achieved deep remission (13/44, 30%), sustained remission (10/34, 29%), remission (8/39, 21%), or LDA (2/39, 5%), *P* < 0.001 for trend (Fig. 1a). These patients were also more likely to maintain DAS28 LDA (31/41, 76%) than those who initially achieved deep remission (16/44, 36%), sustained remission (14/34, 41%), remission (18/39, 46%), or LDA (10/39, 26%), *P* < 0.001. The patients who achieved sustained deep remission in period 1 and then decreased the dose of etanercept to 25 mg were more likely to maintain remission in period 2 (35/43, 81%) than those who achieved deep remission (29/43, 67%), sustained remission (22/38, 58%), remission (20/36, 56%), or LDA (14/39, 36%), *P* < 0.001. The response trend was not significant for maintenance of LDA.

The patients who achieved sustained remission in period 1 based on the ACR/EULAR Boolean criteria and then discontinued etanercept were more likely to maintain remission (10/34, 29%) than those who achieved remission (7/45, 16%) or did not achieve remission (4/119, 3%) in period 1, *P* < 0.001 (Fig. 1b). Patients who decreased the dose had a numerically greater response than those who discontinued; the patients with sustained remission in period 1 were more likely to maintain remission (32/44, 73%) than those who achieved remission (12/32, 38%) or did not achieve remission (23/125, 18%), *P* < 0.001.

In the CDAI analysis, patients in all treatment groups were more likely to maintain remission or LDA in period 2 if they achieved sustained remission in period 1. Additionally, patients who decreased the dose of etanercept had a numerically greater response than those who discontinued etanercept (Fig. 1c). Of the patients who achieved sustained remission in period 1 and then discontinued etanercept, 7/23 (30%) and 19/23 (83%) maintained remission and LDA, respectively, versus 4/33 (12%) and 16/33 (48%) who achieved remission, 11/129 (9%) and 70/129 (54%) who achieved LDA, and 0/11 (0%) and 2/11 (18%) who achieved moderate disease activity, *P* < 0.01 for both. Of the patients who achieved sustained remission in period 1 and then decreased the dose of etanercept, 26/36 (72%) and 33/36 (92%) maintained remission and LDA, respectively, versus 15/30 (50%) and 24/30 (80%) who achieved remission, 23/127 (18%) and 105/127 (83%) who achieved LDA, and 0/8 (0%) and 2/8 (25%) who achieved moderate disease activity, *P* < 0.001 for remission and *P* < 0.05 for LDA.

#### PRIZE study

Patients in the PRIZE study demonstrated significant response trends for all treatment groups according to ACR/EULAR Boolean criteria, and for two treatment groups according to CDAI criteria, with no significant response trends observed according to DAS28 criteria.

**Fig. 2:**
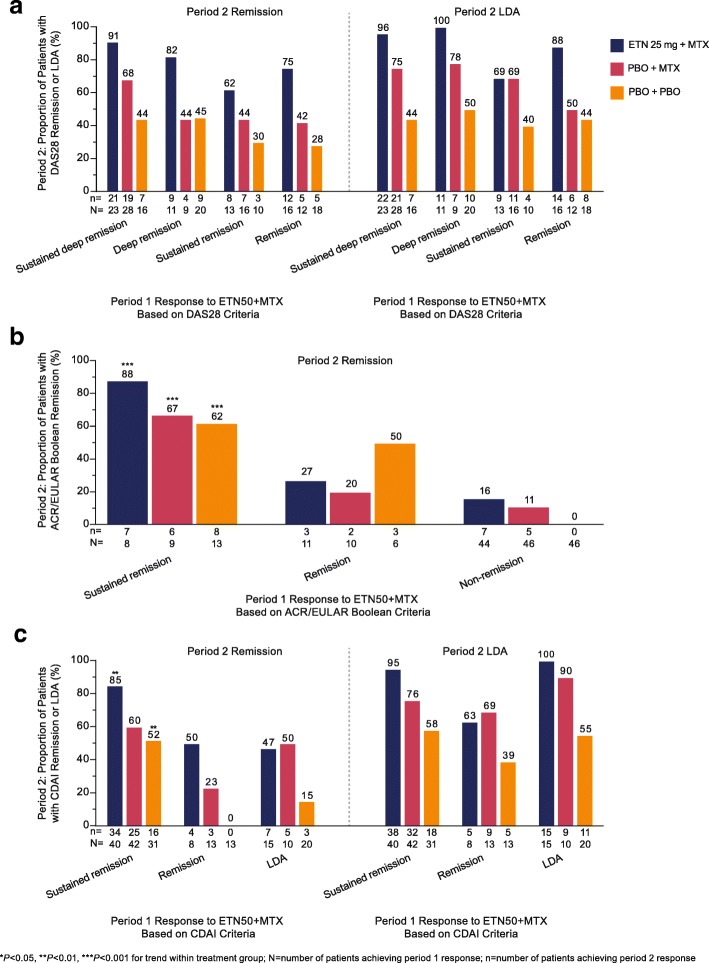
Proportion of patients in the PRIZE study maintaining remission or LDA in period 2, according to response category in period 1 for the remission criteria of DAS28 (**a**), ACR/EULAR Boolean (**b**), and CDAI (**c**). *ACR* American College of Rheumatology, *CDAI* Clinical Disease Activity Index, *DAS28* Disease Activity Score in 28 joints, *ETN50* etanercept 50 mg, *EULAR* European League Against Rheumatism, *LDA* low disease activity, *MTX* methotrexate, *PBO* placebo

In the DAS28 analysis, the group that decreased the dose of etanercept demonstrated a better response than the groups that discontinued etanercept and discontinued etanercept+methotrexate (MTX) (Fig. 2a).

In the ACR/EULAR Boolean remission criteria analysis, some of the sample sizes were small (Fig. 2b). In the group that decreased the dose of etanercept, period 2 remission was maintained by 7/8 (88%) patients with period 1 sustained remission, versus 3/11 (27%) with remission and 7/44 (16%) without remission in period 1, *P* < 0.01. In the group that discontinued etanercept, remission was maintained by 6/9 (67%), 2/10 (20%), and 5/46 (11%), respectively, *P* < 0.001. In the group that discontinued etanercept+MTX, remission was maintained by 8/13 (62%), 3/6 (50%), and 0/46 (0%), respectively, *P* < 0.001.

In the CDAI analysis, a significant response trend was present for the group that decreased the dose of etanercept and the group that discontinued etanercept+MTX (Fig. 2c). In the dose reduction group, period 2 remission was maintained by 34/40 (85%) of patients with sustained remission in period 1 versus 4/8 (50%) and 7/15 (47%) with remission and LDA, respectively, *P* < 0.01. In the group that discontinued etanercept+MTX, period 2 remission was maintained by 16/31 (52%) with sustained remission in period 1, versus 0/13 (0%) and 3/20 (15%) with remission and LDA, respectively, *P* < 0.01.

#### T2T study

**Fig. 3:**
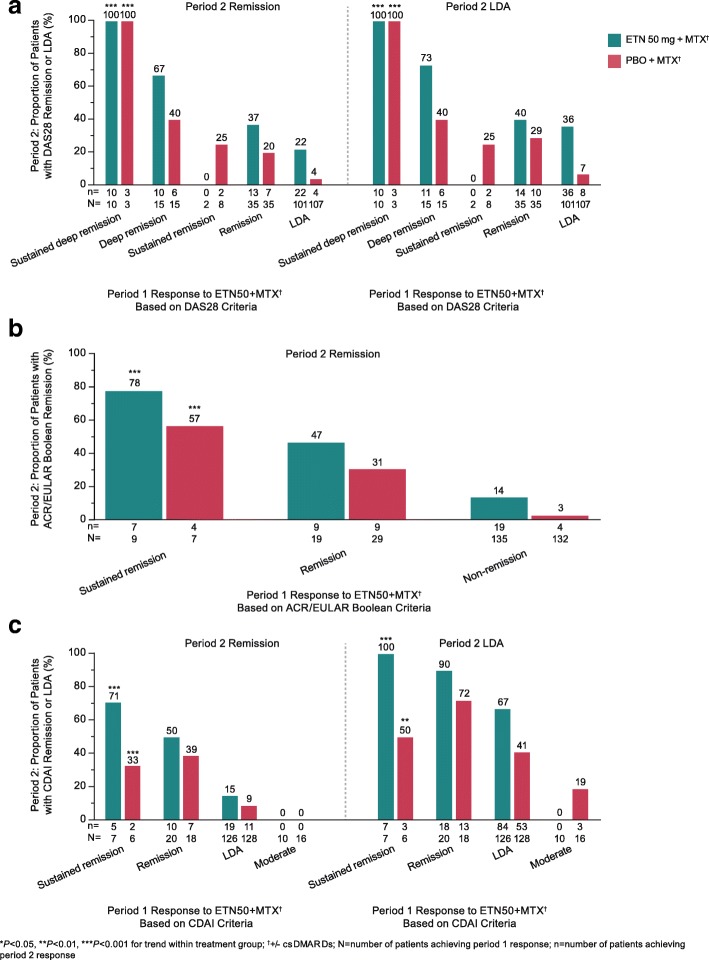
Proportion of patients in the T2T study maintaining remission or LDA in period 2, according to response category in period 1 for the remission criteria of DAS28 (**a**), ACR/EULAR Boolean (**b**), and CDAI (**c**). *ACR* American College of Rheumatology, *CDAI* Clinical Disease Activity Index, *csDMARDs* conventional synthetic disease-modifying antirheumatic drugs, *DAS28* Disease Activity Score in 28 joints, *ETN50* etanercept 50 mg, *EULAR* European League Against Rheumatism, *LDA* low disease activity, *MTX* methotrexate, *PBO* placebo

Patients in the T2T study demonstrated significant response trends in period 2 across all measurement criteria in all treatment groups, although in many instances, the sample sizes were small. In most cases, the patients who continued etanercept 50 mg in period 2 demonstrated higher rates of remission and LDA than those who discontinued etanercept. For the group that discontinued etanercept, DAS28 remission was maintained in period 2 by 3/3 (100%) patients with sustained deep remission in period 1 and by 6/15 (40%), 2/8 (25%), 7/35 (20%), and 4/107 (4%) patients with deep remission, sustained remission, remission, and LDA, respectively, *P* < 0.001 (Fig. 3a). DAS28 LDA was maintained by 3/3 (100%), 6/15 (40%), 2/8 (25%), 10/35 (29%), and 8/107 (7%) patients, respectively, *P* < 0.001.

In the ACR/EULAR Boolean analysis, the proportion of patients who discontinued etanercept and maintained remission was 4/7 (57%), 9/29 (31%), and 4/132 (3%) of the patients with sustained remission, remission, and nonremission in period 1, respectively, *P* < 0.001 (Fig. 3b). In the CDAI analysis, the proportion of patients who discontinued etanercept and maintained remission was 2/6 (33%), 7/18 (39%), 11/128 (9%), and 0/16 (0%) of the patients with sustained remission, remission, LDA, and moderate disease activity in period 1, respectively, *P* < 0.001; LDA was maintained by 3/6 (50%), 13/18 (72%), 53/128 (41%), and 3/16 (19%), *P* < 0.01 (Fig. 3c).

In all three studies, continuing etanercept 50 mg or decreasing the dose to 25 mg resulted in a greater proportion of patients maintaining remission or LDA than discontinuing etanercept.

### Sustained DAS28 deep remission and the Health Assessment Questionnaire

The proportion of patients with a normal HAQ score at the end of period 1 was significantly higher for those patients who achieved sustained DAS28 deep remission at the end of period 1 than for those who did not, in all three studies (Additional file 1: Figure S1). In PRESERVE, 82.0% of patients with sustained DAS28 deep remission had a HAQ score ≤ 0.5, compared with 59.2% of patients who did not achieve sustained DAS28 deep remission, *P* < 0.001. The corresponding values for PRIZE were 94.0% vs 75.0%, *P* = 0.001, and for T2T, 91.7% vs 51.4%, *P* = 0.006.

## Discussion

The results of this analysis demonstrate that overall, the likelihood of patients maintaining remission or LDA following dose reduction or discontinuation of etanercept follows a significant trend, with those initially achieving sustained deep remission being the most likely to maintain a response, followed by those achieving deep remission and sustained remission. This was the case whether remission was measured using DAS28 (except in PRIZE), ACR/EULAR Boolean, or CDAI criteria. Additionally, a greater proportion of patients maintained LDA than remission.

It is of interest to note that most of the remission trend results were similar across the studies, even though the patient populations were different. The PRESERVE and T2T studies represented an established RA population; patients had a disease duration of approximately 7 to 8 years. Conversely, the PRIZE study represented an early RA population with a mean disease duration of 6.5 months. The mean number of prior csDMARDs was slightly higher in the PRESERVE and T2T studies (1.3 for each) than in the PRIZE study (1.0), likely owing to the shorter disease duration in PRIZE. Patients in the PRESERVE and PRIZE studies were bDMARD naïve; only one patient in T2T had a history of bDMARD therapy.

In the PRIZE study, a relatively high proportion of patients achieved sustained deep remission in period 1 compared to the other levels of remission and compared to the other two studies. This result may be related to these patients having early disease, although the PRIZE study also had the longest period of open-label etanercept treatment. In this study only, the response trend according to the DAS28 criteria was not significant, suggesting that in patients with early disease, it may be more appropriate to use the ACR/EULAR Boolean or CDAI remission criteria than the DAS28.

In the HONOR and RRR trials, 80% and 71% of patients with DAS28 deep remission were able to maintain LDA following discontinuation of adalimumab and infliximab, respectively [29, 30]. In the current analysis, the proportions of patients with DAS28 sustained deep remission/deep remission in period 1 who maintained LDA following discontinuation of etanercept were 76%/36%, 75%/78%, and 100%/40% for PRESERVE, PRIZE, and T2T, respectively. Many of these values are similar to those reported in HONOR and RRR [29, 30].

For patients, improvement in functional disability is an important aspect of RA treatment, because it affects daily activities, the ability to work, and long-term morbidity and mortality [41–43]. We found that the proportion of patients with a normal HAQ score at the end of period 1 was significantly higher for those patients who achieved sustained DAS28 deep remission at the end of period 1 than for those who did not, in all three studies. These results are in line with a published study that found that improvement in the HAQ score continues if LDA or remission is achieved and sustained [44]. Another study determined through multivariate analyses that having a lower HAQ score at the start of treatment predicts 2-year sustained DAS28 remission [45]. In our analysis, we did not find HAQ ≤ 0.5 to be among the best subset of significant predictors in maintaining DAS28 remission; however, lower baseline values for HAQ were associated with a better period 1 DAS28 response in PRESERVE and T2T, and a better CDAI response in PRESERVE.

This study has several limitations. The data pertain to etanercept and likely to other tumor necrosis factor (TNF) inhibitors, but not to IL-6 or JAK inhibitors that directly affect the acute-phase response and as a result, may lead to unduly low DAS28 values. Further, the patients included in this study had moderate or moderate-to-severe RA disease activity, so these results may not be applicable to patients with mild or low RA disease activity; however, in these patients, bDMARDs are rarely indicated or reimbursed. In the PRIZE and T2T studies, the sample sizes were small for many of the remission categories at the end of period 1; therefore, the period 2 results need to be interpreted with caution. Also, the duration of exposure to etanercept differed among the three studies, and this is a variable that must be considered when evaluating the results. Additionally, we cannot draw conclusions regarding maintenance of remission or LDA for periods longer than in these studies. Finally, caution should be exercised when generalizing these results to all patients, because the patients in these clinical trials were selected based on strict inclusion and exclusion criteria.

In future studies, it would be interesting to evaluate whether differences between sustained deep remission, deep remission, sustained remission, remission, and LDA are noted on ultrasound. Studies have found that approximately 50–75% of patients with RA in clinical remission still have active synovitis on ultrasound [46–51]. One study that evaluated patients with deep clinical remission (DAS28 ≤ 1.98) and remission (DAS28 ≤ 2.6 but > 1.98) found that significantly fewer patients with deep remission compared to remission had evidence of synovitis on ultrasound [51]. This is in line with using more stringent remission criteria, such as the ACR/EULAR Boolean definition, which does not require definition of “deep” remission, since, as also seen here, it already reflects deep remission.

## Conclusions

The results of this study add to the published evidence that some patients can successfully decrease the dose or discontinue bDMARD therapy [8–16]. In certain cases, this may be desirable for the purposes of decreasing the risk of adverse events and/or reducing costs. Although the most appropriate patients and dose reduction strategy remain unclear, this study found that sustained deep remission, normal ESR, SJC = 0, and TJC = 0 at the end of period 1 were significant predictors of maintaining DAS28 remission after etanercept dose reduction or discontinuation. Additionally, our results suggest that clinicians should consider using a stringent definition of disease control, such as sustained deep remission according to DAS28, or sustained remission according to CDAI/SDAI or ACR/EULAR Boolean criteria. Controlled clinical studies evaluating the endpoints of sustained and/or deep remission are needed.

## Additional file

Additional file 1: **Table S1.** Demographic and baseline disease characteristics according to CDAI response in period 1; **Table S2.** Proportion of patients achieving each ACR/EULAR Boolean criteria response at the end of period 1; **Figure S1.** Proportion of patients with a normal HAQ score at the end of period 1 according to achievement of period 1 sustained DAS28 deep remission (yes/no). (PDF 283 kb)

## Data Availability

Upon request, and subject to certain criteria, conditions, and exceptions (see https://www.pfizer.com/science/clinical-trials/trial-data-and-results for more information), Pfizer will provide access to individual de-identified participant data from Pfizer-sponsored global interventional clinical studies conducted for medicines, vaccines, and medical devices (1) for indications that have been approved in the USA and/or EU or (2) in programs that have been terminated (i.e., development for all indications has been discontinued). Pfizer will also consider requests for the protocol, data dictionary, and statistical analysis plan. Data may be requested from Pfizer trials 24 months after study completion. The de-identified participant data will be made available to researchers whose proposals meet the research criteria and other conditions, and for which an exception does not apply, via a secure portal. To gain access, data requestors must enter into a data access agreement with Pfizer.

## Abbreviations

ACR: American College of Rheumatology
APLAR: Asia Pacific League of Associations for Rheumatology
bDMARD: Biologic disease-modifying antirheumatic drug
BMI: Body mass index
CDAI: Clinical Disease Activity Index
CMH: Cochran-Mantel-Haenszel
CRP: C-reactive protein
csDMARD: Conventional synthetic DMARD
DAS28: Disease Activity Score 28-joint count
ESR: Erythrocyte sedimentation rate
ETN: Etanercept
EULAR: European League Against Rheumatism
HAQ: Health Assessment Questionnaire
IL: Interleukin
JAK: Janus kinase
LDA: Low disease activity
MTX: Methotrexate
PBO: Placebo
PGA: Physician global assessment
QW: Weekly
RA: Rheumatoid arthritis
SDAI: Simplified Disease Activity Index
SJC: Swollen joint count
T2T: Treat-to-Target
TJC: Tender joint count
TNF: Tumor necrosis factor
ULN: Upper limit of normal
