# Supplementary material for: The effect of deep or sustained remission on maintenance of remission after dose reduction or withdrawal of etanercept in patients with rheumatoid arthritis
Source: Arthritis Res Ther. 2019 Jul 5;21:164. doi: 10.1186/s13075-019-1937-4 (PMC6610967; doi:10.1186/s13075-019-1937-4)
Supplement: Supplementary file 1 — Table S1. Demographic and baseline disease characteristics according to CDAI response in period 1; Table S2. Proportion of patients achieving each ACR/EULAR Boolean criteria response at the end of period 1; Figure S1. Proportion of patients with a normal HAQ score at the end of period 1 according to achievement of period 1 sustained DAS28 deep remission (yes/no). (PDF 283 kb) [file 13075_2019_1937_MOESM1_ESM.pdf]

Additional file 1

Table S1 Demographic and baseline disease characteristics according to CDAI response at the end of period 1

	PRESERVE Study, N=598				PRIZE Study, N=192			T2T Study, N=331			
	Sustained remission n=99 (17%)	Remission n=97 (16%)	LDA n=368 (62%)	MDA n=34 (6%)[a]	Sustained remission n=113 (59%)	Remission n=34 (18%)	LDA n=45 (23%)	Sustained remission n=13 (4%)	Remission n=38 (11%)	LDA n=254 (77%)	MDA n=26 (8%)
Demographic characteristics											
Age, y, mean (SD)	44.6 (13.3)	45.0 (11.7)	48.6 (11.6)	52.6 (12.8)***	47.0 (14.5)	49.1 (12.3)	55.6 (14.3)***	42.9 (12.2)	41.5 (14.5)	47.1 (11.8)	51.4 (12.5)**
Female, n (%)	80 (81)	81 (84)	297 (81)	24 (71)	71 (63)	20 (59)	33 (73)	10 (77)	34 (90)	217 (85)	18 (69)
BMI, kg/m^2, mean (SD)	25.4 (4.2)	24.4 (3.7)	25.9 (4.9)	25.5 (3.6)	25.2 (4.5)	26.8 (5.0)	26.6 (4.3)	28.0 (5.1)	26.0 (5.4)	27.0 (5.5)	26.7 (4.8)
BMI, female	25.0 (3.8)	24.3 (3.7)	25.8 (5.1)	25.9 (3.6)*	24.1 (4.1)	25.3 (5.2)	26.1 (4.3)*	28.4 (5.2)	25.9 (5.5)	27.0 (5.5)	26.3 (5.3)
BMI, male	27.1 (5.2)	25.1 (3.5)	26.2 (4.1)	24.8 (3.7)	27.0 (4.7)	28.9 (4.1)	28.0 (3.9)	26.5 (5.4)	26.8 (4.8)	27.0 (5.4)	27.4 (4.0)
Prior treatment[b]											
Corticosteroid(s), n (%)	47 (47)	62 (64)	225 (61)	24 (71)*	35 (31)	21 (62)	22 (49)*	8 (62)	26 (68)	176 (69)	14 (54)
NSAID(s), n (%)	75 (76)	72 (74)	276 (75)	26 (76)	77 (68)	27 (79)	27 (60)	9 (69)	25 (66)	170 (67)	16 (62)
DMARD(s),[c] n (%)	23 (23)	23 (24)	90 (24)	14 (41)	18 (16)	8 (24)	7 (16)	4 (31)	14 (37)	94 (37)	7 (27)
Number of DMARD(s),[c,d] mean (min, max)	1.5 (1, 5)	1.2 (1, 2)	1.2 (1, 3)	1.3 (1, 2)	1.0 (1, 1)	1.0 (1, 1)	1.0 (1, 1)	1.3 (1, 2)	1.4 (1, 3)	1.3 (1, 3)	1.1 (1, 2)
Disease characteristics											
Duration of disease, mean (SD)	6.5 (6.5) y	6.4 (6.5) y	7.0 (7.3) y	7.6 (6.5) y	6.7 (2.9) mos	7.4 (2.9) mos	6.9 (2.6) mos	6.4 (5.4) y	5.8 (5.6) y	8.7 (7.2) y	7.2 (7.8) y

RF+, n (%)	75 (76)	70 (72)	265 (72)	23 (68)	74 (65)	16 (47)	22 (49)*	11 (85)	32 (84)	213 (84)	19 (73)
aCCP antibody+, n (%)	77 (78)	71 (73)	297 (81)	25 (74)	81 (72)	22 (65)	24 (53)*	10 (77)	28 (74)	205 (81)	20 (77)
ESR, mm/h, mean (SD)	25.5 (15.8)	21.4 (10.8)	21.1 (12.4)	13.7 (9.3)***	36.0 (24.0)	31.7 (18.4)	28.5 (17.2)*	43.3 (18.0)	53.2 (28.4)	47.1 (25.6)	33.4 (15.3)
CRP, mg/L, mean (SD)	12.2 (15.1)	10.4 (13.5)	11.9 (15.5)	12.5 (17.5)	17.3 (24.5)	15.7 (19.5)	11.5 (13.0)	27.6 (16.5)	21.0 (17.3)	22.6 (27.1)	25.1 (27.4)
Disease activity and patient-reported outcomes, mean (SD)											
TJC (0-28)	4.4 (2.1)	4.7 (3.0)	5.2 (2.9)	5.7 (3.7)**	12.3 (6.4)	14.1 (6.1)	15.4 (7.0)**	14.3 (7.9)	14.0 (6.7)	13.7 (6.4)	15.5 (5.0)
SJC (0-28)	3.6 (2.1)	3.3 (1.7)	4.0 (2.7)	6.1 (4.3)***	9.7 (5.8)	10.3 (5.2)	11.8 (5.2)*	12.6 (5.4)	9.9 (5.3)	10.4 (5.5)	11.4 (5.6)
DAS28-ESR, mean (SD)	4.3 (0.5)	4.2 (0.5)	4.4 (0.4)	4.2 (0.5)	5.7 (1.1)	5.9 (1.0)	6.0 (1.0)	6.4 (1.2)	6.4 (1.0)	6.4 (1.0)	6.3 (0.7)
CDAI (0-76)	16.4 (4.1)	16.3 (5.0)	18.1 (4.9)	21.5 (6.2)***	–	–	–	39.5 (13.9)	37.3 (12.6)	37.6 (11.9)	39.4 (10.2)
PGA (0-10)	3.9 (1.3)	3.8 (1.3)	4.1 (1.3)	4.4 (1.5)*	5.4 (1.6)	5.6 (1.7)	5.6 (1.5)	6.2 (1.4)	6.9 (1.7)	6.8 (1.3)	6.5 (1.4)
HAQ (0-3)	1.0 (0.6)	1.0 (0.6)	1.1 (0.6)	1.2 (0.6)*	1.2 (0.6)	1.3 (0.7)	1.2 (0.7)	1.4 (0.8)	1.5 (0.7)	1.5 (0.6)	1.4 (0.5)

[a]Sum of percentages is greater than 100 due to rounding.
[b]In PRESERVE, “prior” was within 6 months of screening (for DMARDs not including MTX), within 28 days of screening or baseline (for glucocorticoids), or concurrent treatment with ≥1 NSAID at baseline; in PRIZE, “prior” was 28 days before screening (for NSAIDs and corticosteroids) and any time before screening (for DMARDs); in T2T, “prior” was 6 months before screening (for MTX and DMARDs,) or 4 weeks before screening (for NSAIDs and corticosteroids).
[c]Conventional DMARDs other than MTX.
[d]Mean, minimum, and maximum were calculated only from patients who did have prior DMARD(s).

*$P < 0.05$ across response categories; **$P < 0.01$ across response categories; ***$P < 0.001$ across response categories.

aCCP anti-cyclic citrullinated peptide, *BMI* body mass index, *CDAI* Clinical Disease Activity Index, *CRP* C-reactive protein, *DAS28* Disease Activity Score in 28 joint, *DAS28-ESR* Disease Activity Score in 28 joints calculated with erythrocyte sedimentation rate, *DMARD* disease-modifying antirheumatic drug, *ESR* erythrocyte sedimentation rate, *HAQ* health assessment questionnaire, *LDA* low disease activity, *MTX* methotrexate, *NSAID* nonsteroidal anti-inflammatory drug, *PGA* physician global assessment, *RA* rheumatoid arthritis, *RF* rheumatoid factor, *SD* standard deviation, *SJC* swollen joint count, *TJC* tender joint count.

Table S2. Proportion of patients achieving each ACR/EULAR Boolean criteria response at the end of period 1

	Sustained remission n (%)	Remission n (%)	Non-remission n (%)
PRESERVE, N=600	123 (21)	121 (20)	356 (59)
PRIZE, N=193	30 (16)	27 (14)	136 (70)
T2T, N=331	16 (5)	48 (15)	267 (81)[a]

[a]Sum of percentages is greater than 100 due to rounding.

Fig. S1 Proportion of patients with a normal HAQ score at the end of period 1 according to achievement of Period 1 sustained DAS28 deep remission (yes/no).

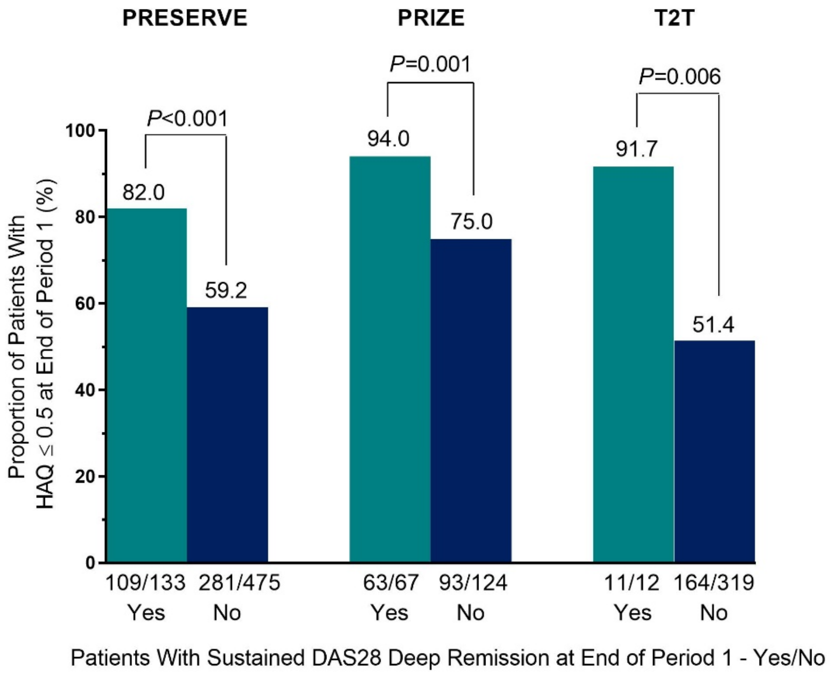

DAS28 disease activity score-28 joints, *HAQ* Health Assessment Questionnaire.
